# The Effect of PCSK9 Inhibition on the Stabilization of Atherosclerotic Plaque Determined by Biochemical and Diagnostic Imaging Methods

**DOI:** 10.3390/molecules28155928

**Published:** 2023-08-07

**Authors:** Marcin Basiak, Marcin Hachula, Michal Kosowski, Grzegorz Machnik, Mateusz Maliglowka, Maria Dziubinska-Basiak, Robert Krysiak, Boguslaw Okopien

**Affiliations:** 1 Department of Internal Medicine and Clinical Pharmacology, Medical University of Silesia, Medyków 18, 40-752 Katowice, Poland; 2 SCANiX Medical Imaging, Ceglana 35, 40-514 Katowice, Poland

**Keywords:** PCSK-9 inhibitors, atherosclerotic plaque, carotid MRI, osteopontin, osteoprotegerin, matrix metalloproteinases

## Abstract

Atherosclerosis is a multifactorial, progressive, chronic inflammatory disease. Ultrasound and magnetic resonance imaging are the most accurate predictors of atherosclerotic plaque instability (MRI). Cytokines such as osteopontin, osteoprotegerin, and metalloproteinase 9 could be used as the most recent markers to identify and track the efficacy of anti-atherosclerotic therapy. Patients with USG and MRI-verified unstable atherosclerotic plaque were included in the study. Biomarker concentrations were measured and compared before and after PCSK9 inhibitor therapy. Additionally, concentrations prior to treatment were correlated with MRI images of the carotid artery. After treatment with alirocumab, the concentrations of MMP-9 (*p* < 0.01) and OPN, OPG (*p* < 0.05) decreased significantly. Furthermore, the results of OPN, OPG, and MMP 9 varied significantly depending on the type of atherosclerotic plaque in the MRI assay. In stable atherosclerotic plaques, the concentrations of OPN and OPG were greater (*p* < 0.01), whereas the concentration of MMP9 correlated with the instability of the plaque (*p* < 0.05). We demonstrated, probably for the first time, that alirocumab therapy significantly decreased the serum concentration of atherosclerotic plaque markers. In addition, we demonstrated the relationship between the type of atherosclerotic plaque as determined by carotid MRI and the concentration of these markers.

## 1. Introduction

Cardiovascular diseases (CVD), such as coronary artery disease (CAD), peripheral artery disease (PAD), and cerebrovascular disease, are one of the leading causes of death in industrialized societies and a worldwide concern. Atherosclerosis, a multifactorial, chronic, progressive inflammatory disease that affects the arteries throughout the body [1,2], is the leading cause of cardiovascular disease. Numerous studies have demonstrated that inflammation plays a crucial role in atherogenesis, as indicated by the presence of a large number of inflammatory cells, primarily monocytes, macrophages, and T lymphocytes in the atherosclerotic plaque [3,4]. Atherogenesis begins with the accumulation of plasma lipoproteins, particularly low-density lipoprotein cholesterol (LDL-C), in the endothelium, the activation of inflammatory cells, and the increase in collagen synthesis in vascular smooth muscle cells. It is followed by the production of proinflammatory cytokines, hydrolytic enzymes, coagulation factors, and adhesion molecules by activated macrophages and T cells. They may play a role in the progression and instability of atherosclerotic plaques [4,5,6,7]. High-risk or vulnerable plaques are associated with an increased risk of plaque rupture, embolism not under control, and cardiovascular events. Changes in the plaque’s histological structure reveal that it has a large lipid core, a thin fibrin-coated cover, wall clots or effusions within, and a high number of macrophages and other inflammatory cells. Additionally, this type of plaque may exhibit ulceration [8,9]. Imaging techniques such as ultrasonography and highly sensitive and specific magnetic resonance imaging (MRI) are the most accurate at predicting when an atherosclerotic plaque will begin to disintegrate [10,11]. Numerous previous studies [12,13,14,15] indicate that standard lipid-lowering therapy with statins reduces intravascular lesions as measured by intravascular ultrasound (IVUS). Not only is the inflammatory process detected in arterial plaque but also in peripheral blood. Blood levels of inflammatory markers may be useful for estimating cardiovascular risk and tracking disease progression. Serum concentrations of C-reactive protein (CRP), interleukin-6 (IL-6), and LDL are highly correlated with clinical symptoms of atherosclerosis and an increased risk of death from cardiovascular disease [16,17].

In the last century, medical imaging played a key role in assessing carotid arteries. They allow visualization of the atherosclerosis plaque. The precision of recognizing the pathology state exceeds 90% [18,19]. Magnetic resonance imaging is currently the most sensitive method of diagnosing atherosclerotic lesions [10]. Carotid plaque imaging techniques by MRI include black-blood and bright-blood imaging. Black-blood imaging is a technique that uses double or quadruple inversion recovery in T1, T2, and proton density sequences to suppress the signal from circulating blood. The lumen appears dark, allowing for a more precise delineation of the vessel wall and plaque components. Bright-blood is a technique for magnetic resonance angiography (in which the lumen appears to be hyperintense); it utilizes gradient-recalled echo sequences (e.g., 3D time-of-flight). This sequence is advantageous for enhancing the visibility of the fibrous cap and superficial calcifications. The current recommendation for imaging and interpretation of carotid plaques is the use of various contrast image sequences: T1, T2, proton density, 3D time-off light, and postcontrast T1 sequences [20]. Gadolinium decreases the T1 time constant and increases contrast resolution and signal-to-noise ratio (SNR) [21]. It results in a more defined fibrous cap and lipid core, higher fibrous tissue intensity, and is associated with neovascularization and macrophage infiltration. To minimize motion artifacts, all MRI sequences are fat-suppressed and cardiac-triggered. MRI can be used to define the form of carotid plaques as well as to identify and quantify plaque components such as the lipid-rich necrotic core, fibrous cap thickness, intraplaque hemorrhage (IPH), and calcifications. Multi-contrast MRI (T1, T2, proton density, and 3D time-of-flight) had a sensitivity of 81% and a specificity of 90% for detecting a thin or ruptured cap. The thickness of the fibrous cap on T1-weighted (T1W) imaging, both pre-and post-gadolinium injection, also correlates well with histology [22]. A retrospective investigation indicated an increased risk of developing symptoms in patients with a previously detected burst fibrous cap by MRI, while a prospective analysis discovered an increased risk of developing symptoms in patients with a previously detected ruptured fibrous cap by MRI [23]. Multi-contrast MRI correctly found IPH as a hyperintense signal on T1W turbo spin-echo pictures with a sensitivity of 93% and a specificity of 96% and on T1W 3D gradient echo images (direct thrombus MRI) with a sensitivity and specificity of 84% and 86%, respectively [24]. There is a substantial correlation between IPH and cerebral ischemia symptoms in retrospective and prospective investigations. IPH was related to considerable plaque progression during an 18-month period in asymptomatic patients [25]. Parmar et al. reported that type VI plaque was associated with an increased risk of cerebral ischemia, which proves its instability [26].

However, researchers worldwide are constantly looking for other biomarkers to detect vulnerable plaque, which could be used in the prevention of cardiovascular episodes and also as markers used in monitoring the response to anti-atherosclerotic therapy. Examples of these biomarkers are osteopontin (OPN), osteoprotegerin (OPG), soluble CD40 ligand (sCD40L), metalloproteinase 9 (MMP-9), and myeloperoxidase (MPO). Myeloperoxidase (MPO) is a protein that belongs to the family of heme peroxidases and is mainly expressed in neutrophils and monocytes. MPO plays a major role in antimicrobial activity against various pathogens, mainly by participating in phagocytosis. Elevated MPO plasma levels are strongly related to inflammation reactions and increased cellular oxidative stress [27,28]. Multiple studies show a strong correlation between MPO and CVD, including CAD and PAD [29,30,31]. Matrix metalloproteinases (MMPs) are zinc-dependent enzymes involved in extracellular matrix remodeling and are responsible for leukocyte recruitment to inflammatory spots, thus acting as important regulators of the inflammation process. Therefore, excessive or imbalanced MMP-9 secretion is related to tissue damage in several inflammatory disorders [32]. MMP-9 plays a part in the progression of arteriosclerosis. MMPs are detected in atherosclerotic plaques and in peripheral blood. Many studies show that plasma levels of MMP-9 are associated with a high risk of plaque rupture and recurrent cardiac events [33,34]. CD40 is a protein situated on the surface of antigen-presenting cells that is activated by T-cell CD40 ligand. This interaction promotes the release of various inflammatory cytokines due to the activation of endothelial cells [35]. Studies show a correlation between the CD40/CD40L signaling pathway and the development and progression of atherosclerosis [36]. OPG is a soluble glycoprotein belonging to the TNF- α (tumor necrosis factor α) receptor family with pleiotropic effects on bone metabolism. It was originally discovered as a bone resorption inhibitor, and its expression and production are regulated by various cytokines and hormones. Recently, studies have shown that OPG is produced in vitro by a variety of tissues, including smooth muscle cells and endothelial cells [37]. In patients with CAD, there is a link between OPG levels and inflammation in the arterial wall [38]. OPN is a phosphorylated glycoprotein found mainly in bone tissue and is responsible for bone formation and calcification. Studies showed OPN to be a multifunctional protein that is upregulated in different inflammatory conditions, including atherosclerosis, and is responsible for the calcification process of plaques [38]. Moreover, many reports prove the correlation between plasma OPN levels and the severity of the narrowing of the arteries underlain by CVD [39].

Protein convertase subtilisin/kexin type 9 (PCSK9) inhibitors are the newest class of cholesterol-lowering drugs. The effect of PCSK9 is a decrease in the number of LDL receptors (LDL-R). PCSK9 inhibitors act by inhibiting the circulating PCSK9, thereby increasing LDL-R levels, which promotes the uptake of LDL by the liver and leads to a reduction in LDL-C concentration in serum. In accordance with the guidelines of the American Heart Association (AHA) and the European Society of Cardiology (ESC), they are used as monotherapy or in combination with statins or ezetimibe to intensify lipid-lowering therapy [40,41]. Recently, it was discovered that the lipid-lowering effect is not the only effect of action that PCSK9 inhibitors have. Pleiotropic properties such as anti-atherosclerotic, plaque stabilization, anti-aggregation, anti-coagulant effect, and inflammation reduction were described [42,43]. Several epidemiological studies have explored the relationship between PCSK9 and inflammation. These studies examined the relationship between PCSK9 and a number of conventionally important inflammatory markers, including white blood cells (WBCs), fibrinogen, and hs-CRP (Table 1).

Despite the fact that PCSK9 inhibitors are relatively new drugs available on the market, their safety profile has been quite well known, and the results of clinical trials and meta-analyses clearly indicate that they are well-tolerated drugs [48,49,50,51]. A meta-analysis of 25 RCTs showed only 1.9% of patients on evolocumab therapy discontinued therapy due to severe adverse events, compared to 4.8% for aliroocumab in the long-term follow-up [52]. In a meta-analysis of 39 RCTs, including a total of 66,478 patients, Guedeney P et al. found no difference between placebo and PCSK9 inhibitors for neurocognitive impairment, rhabdomyolysis, or new-onset type 2 diabetes [53]. The most frequently reported side effects were gastrointestinal upset, upper respiratory tract infections, and musculoskeletal complaints, which were most often resolved during follow-up [54].

A new drug with a common mechanism of action—blocking the PCSK9 protein—inclisiran is a small interfering RNA (siRNA). The main action of siRNA is post-transcriptional gene silencing. In addition to reducing LDL-C, inclisiran also reduces the level of atherogenic lipoprotein A (LpA), reducing cardiovascular risk [55]. This effect was not observed for statins and ezetimibe; the first lipid-lowering drugs for which it was demonstrated were PCSK9 inhibitors. Over 50% reduction of LDL-C was demonstrated during inliciran therapy [56]. Most of the reported adverse effects of inclisiran were mild and disappeared during observation; they were limited to injection site reactions. Other systemic side effects were observed, like infectious symptoms, e.g., musculoskeletal pain, fever, fatigue, or nasopharyngitis [57]. Due to its specific mechanism of action, inclisiran can be used in patients with chronic kidney disease or liver damage [58].

The aim of the study was to assess the effect of PCSK9 inhibitors in a 90-day intervention on the serum levels of arteriosclerotic plaque markers such as sCD40L, OPN, OPG, MMP 9, and MPO and the correlation between marker concentration and the results of a carotid MRI examination.

## 2. Results

### 2.1. Comparison between the Study and the Control Group

There were no observed significant differences in terms of demographic data (age, gender, smoking, and weight) between the study and control group (Table 2).

However, statistically significantly lower concentrations of total cholesterol (TC), LDL-C, high-density lipoprotein cholesterol (HDL), and triglycerides (TG) (*p* < 0.001) were observed in the control group. The concentrations of the tested arteriosclerotic markers between the control group and the study group before treatment were compared. We observed that in the control group, concentrations of OPN (*p* < 0.01), OPG (*p* < 0.01), MMP-9 (*p* < 0.05) were statistically significantly lower. There were no statistically significant differences in sCD40L and MPO concentrations between these two groups (Table 3).

### 2.2. Changes in Serum Biomarkers

**Figure 1:**
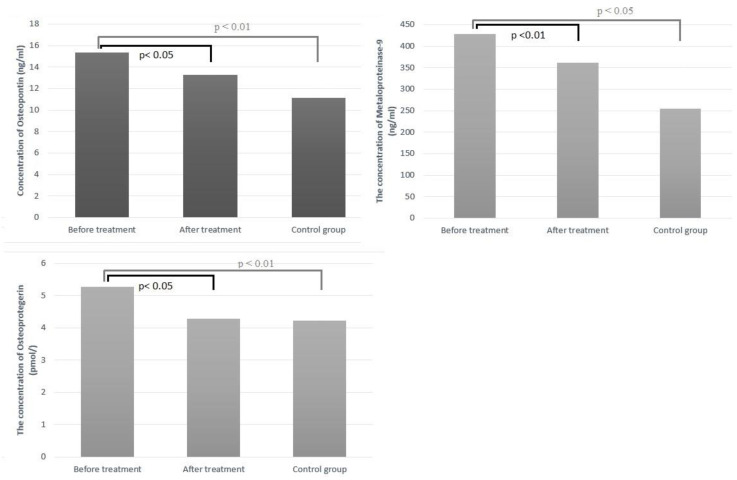
The concentration of atherosclerotic markers depends on the group.

The effect of treatment with PCSK-9 inhibitors on the concentrations of the individual mediators mentioned above was estimated. There was observed, after treatment, a statistically significant decrease in concentrations of MMP-9 (*p* < 0.01) and OPN, OPG (*p* < 0.05). In order, there were no statistically significant differences in sCD40L and MPO concentrations before and after treatment (Table 4). The concentration of arteriosclerotic markers that were statistically significant depending on the subject group is presented in Figure 1.

### 2.3. Correlation of Concentrations of Arteriosclerotic Markers with Results of Carotid Artery MRI Examination

We observed a significant difference in the results of OPN, OPG, and MMP 9 depending on the type of atherosclerotic plaque in the MRI assay. In atherosclerotic plaques considered more stable, the levels of OPN and OPG were higher (*p* < 0.01), while the levels of MMP9 concentration correlated with the instability of the atherosclerotic plaque (*p* < 0.05) (Table 5).

## 3. Discussion

Atherosclerosis contributes to the onset and progression of cardiovascular disease. CVD is the most common cause of death worldwide [59]. The group of cardiovascular diseases includes stroke. It is at the top of the list of causes of death. Each year, 12.2 million patients worldwide suffer a cerebral infraction, of which 62.4% are ischemic. It is estimated that this issue will increase by a factor of two over the next decade. Approximately 87% of ischemic strokes are attributed to modifiable risk factors, such as lipid levels [60]. One of the most common underlying causes of stroke [61] is atherosclerotic disease, which typically affects the proximal portion of the internal carotid arteries. Today, the healthcare system faces challenges in terms of early diagnosis, early identification of pathological conditions that may lead to this condition, and implementation of the appropriate treatment. Scientists from all over the world were interested in a project aimed at discovering new, simple diagnostic methods for atherosclerosis.

To the best of our knowledge, we assessed for the first time the appearance of atherosclerotic plaque using carotid magnetic resonance imaging in relation to plasma levels of plaque markers such as OPN, OPG, MMP-9, MPO, and sCD40l. We showed a statistically significant higher level of OPG and OPN serum concentration in patients with atherosclerotic plaques classified according to AHA in the MRI study as less unstable than VI, containing possible calcification (types IV and V) [23]. Conversely, a higher level of MMP-9 was present in patients with type VI atherosclerotic plaque, which according to the AHA is described as unstable, containing a thrombus, and having intraplaque hemorrhage. There was no correlation between the MRI examination and the concentrations of MPO and sCD40l.

In our work, the biochemical levels of atherosclerosis markers OPN, OPG, and MMP-9 showed statistically significant differences between the study group and healthy people in the control group. In vivo overexpression of OPN in mice caused intensified atherosclerotic plaque formation [62]. In our work, we proved that in patients with carotid atherosclerotic plaque, the serum concentration of OPG was statistically significantly higher than in the control group. A former study showed that serum OPN concentration is associated with early carotid atherosclerosis [63]. Additionally, Golledge et al. observed significantly higher OPN expression in carotid plaque after the endarterectomy of symptomatic patients [64]. The results of our study showed that a higher level of OPN concentration was present in patients whose atherosclerotic plaque was more stable on MRI (Type IV and V according to the AHA) [65]. Similar results have been reported by Polonskaya Y et al. They showed that OPN levels are lower in unstable plaques in patients who undergo endarterectomy during coronary bypass surgery [66]. In turn, findings by Kadoglou et al. suggest the notion that OPN down-regulates plaque calcification and may promote plaque instability as assessed by Gray-scale and color Doppler ultrasound examination [67]. Additionally, patients with acute coronary syndrome had a higher serum concentration of OPN than patients with stable coronary artery disease [68]. It could be said with full conviction that OPN is involved in the process of developing atherosclerotic plaque [69]. According to some studies, elevated serum OPG concentrations are associated with an increase in morbidity and mortality from cardiovascular disease [70,71]. A prospective study showed that osteoprotegerin is responsible for the progression of atherosclerosis in carotid arteries and an increased incidence of cardiovascular disease [65,67,72,73]. OPG levels have been linked to an increased risk of fatal strokes [74]. This action remains unknown. On the other hand, animal models showed that mice lacking the OPG gene developed atherosclerosis much faster [75]. In our survey, we proved that in patients with carotid atherosclerotic plaque, the serum concentration of OPG was statistically significantly higher than in the control group. Similar results were reported by Abedin et al., who reported OPG levels were significantly higher in subjects with atherosclerosis in the aorta in their study, which was performed on 2392 subjects [76]. In addition, Dekker had analogous observations on 742 patients at a coronary artery calcium CT examination in symptomatic patients [77]. In a prospective study of patients with acute myocardial infarction, Cottin et al. announced an association between OPG concentration and the intensity of CAD estimated by the SYNTAX scale. A recent study showed high levels of OPG are independently associated with major damage to the myocardium after ST-elevated myocardial infarction (STEMI) [78]. A recent meta-analysis confirmed that higher levels of OPG are linked to a higher risk of death from all causes and heart disease in people with chronic kidney disease [79,80,81]. We showed a correlation between more calcified atherosclerotic plaque by MRI examination and a greater serum OPG concentration. A similar observation was made by Strobescu-Ciobanu in the study on patients with atherosclerosis confirmed by USG examination and histologically assessed specimens after carotid endarterectomy. Their study proved that OPG is strongly expressed in stable calcified plaques [82].

Macrophages and the metalloproteinase-9 (MMP-9) play a crucial role in the transformation of an atherosclerotic plaque into an unstable one, particularly by weakening the fibrous cap of the plaque [83]. A previous study demonstrated that patients with ruptured plaques had elevated serum levels of MMP-9, and that MMP-9 was an independent risk factor for plaque rupture [84]. In patients with carotid stenosis, elevated serum accumulation of matrix metalloproteinase-9 was associated with doubling the risk of stroke [85]. Olson et al. did not find a correlation between plasma MMP-9 concentration and the presence of atherosclerotic plaque in the carotid artery [86]. We observed a higher level of MMP-9 in a group with potentially rupture-prone atherosclerotic plaque type VI as determined by MRI. Similar outcomes were achieved. Tan et al. found a correlation between elevated serum MMP-9 concentrations and plaque instability, as measured by ultrasound [87].

It is a known fact that higher PCSK9 plasma levels have been linked with atherosclerosis progression via various mechanisms dependent on lipoprotein and also a pro-inflammatory state in plasma connected to circulating chemokines and cytokines [88,89]. It seems no studies have been performed so far that show the direct effect of PCSK-9 inhibitors on the concentrations of markers of plaque vulnerability like MMPs, OPN, and OPG, but other studies support the hypothesis that PCKS-9 inhibitors have a stabilizing effect on atherosclerotic plaque. Results from a single-arm mechanistic study showed that after 6 months of PCSK9 inhibition with alirocumab, carotid plaque lipid content was lower by 17% as assessed by MRI [90]. Additionally, the randomized controlled trial with evolocumab proved a beneficial effect on the reduction of atherosclerotic plaques, as assessed using IVUS [91]. There is evidence that the inhibition of PCSK9 improves coronary endothelial function through non-invasive MRI methodology [92]. Moreover, a study conducted by Otake et al. describes the positive effect of therapy with alirocumab on the atherosclerosis plaque vulnerability assessed with optical coherence tomography [93]. In our study, after treatment with alirocumab, a decrease in concentrations of MMP-9, OPN, and OPG was observed to be statistically significant. We were probably the first to describe this relationship in vivo. However, a similar conclusion in animal models was reached by Elsweid et al., who reported that polyconasol, a drug with an influence on lowering serum PCSK-9 concentration, reduces OPN levels [94].

Our research has a few limitations. The major limitation is the small size of the control group, but this is a pilot study that serves as a foundation for future investigation. The lack of a placebo group represents the second major limitation. This is because it would be unethical to delay PCSK-9 inhibitor treatment in this group of patients, who are at a very high risk of CVD mortality. MRI follow-up of alirocumab-treated patients could add value to this study. The short follow-up period of only three months may also be a significant limitation.

## 4. Materials and Methods

**Figure 2:**
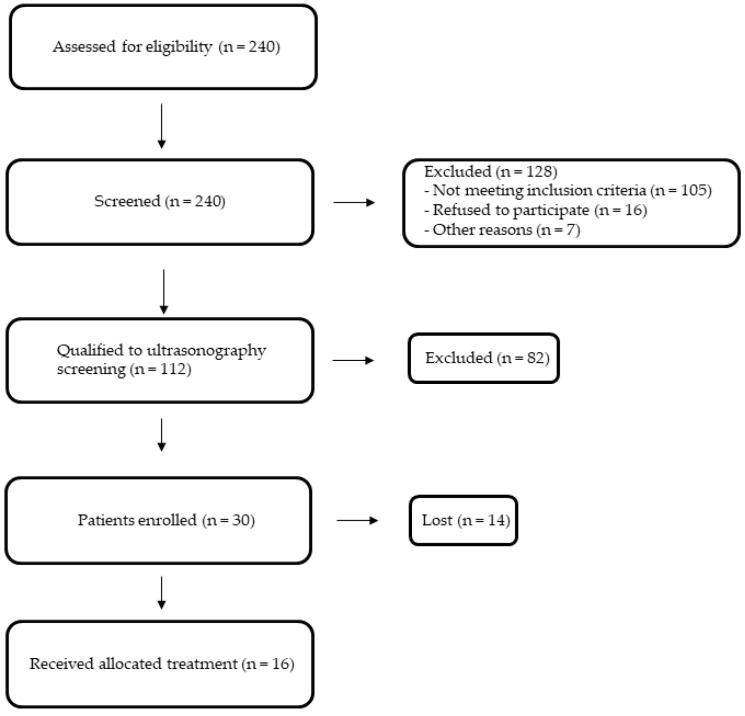
Study flow-chart.

The medical experiment was performed in the years 2019–2020. In our study, we enrolled 16 patients (all of them were our clinical department patients), mean age of 58 +/− 6 years, diagnosed with dyslipidemia, and assessed unstable atherosclerosis based on B-mode ultrasound common carotid intima-media thickness. The method of qualifying patients for the study is shown in Figure 2.

Subjects who fulfilled all the very detailed and narrow inclusion criteria were eligible for study entry. In the control group, there were 12 participants who were matched by age and sex, and all were healthy people. Each patient gave their informed consent in accordance with the Declaration of Helsinki. All the information about the subjects was anonymized. The study protocol was approved by the Bioethical Committee of the Medical University of Silesia PCN-1-185/N/9/O 2019. All included subjects were treated with a constant dose of alirocumab (150 mg) administered every two weeks at the same time of day for 90 days.

### 4.1. Inclusion and Exclusion Criteria

Entry criteria were mixed dyslipidemia (former Frederickson hyperlipidemia type 2B)—plasma TC > 200 mg/dL, LDL > 135 mg/dL, TG > 150 mg/dL, with recognized arterial hypertension and the presence of atherosclerotic plaque in the common carotid artery confirmed by USG examination. Patients were excluded from the study in the case of other types of dyslipidemias, as well as secondary causes of dyslipidemia in the course of thyroid diseases; chronic pancreatitis; autoimmune disorders; nephrotic syndrome; liver and biliary tract diseases; obesity (body mass index > 30 kg/m^2^); alcoholism; any acute and chronic inflammatory processes; treatment for infection; cardiac disorders like exacerbation of chronic heart failure and unstable coronary artery disease; myocardial infarction or stroke in past medical history; treatment with other hypolipidemic drugs (statins, fibrates, and ezetimibe) within 3 months before the study; simultaneous treatment with other drugs that affect plasma lipid levels (i.e., polyunsaturated fatty acids, monacolin K); and concomitant treatment with drugs that may affect inflammatory processes in the vascular wall (including nonsteroid anti-inflammatory drugs and angiotensin-converting enzyme inhibitors) within 3 months of the enrollment.

### 4.2. Arteriosclerotic Plaque Examination

**Figure 3:**
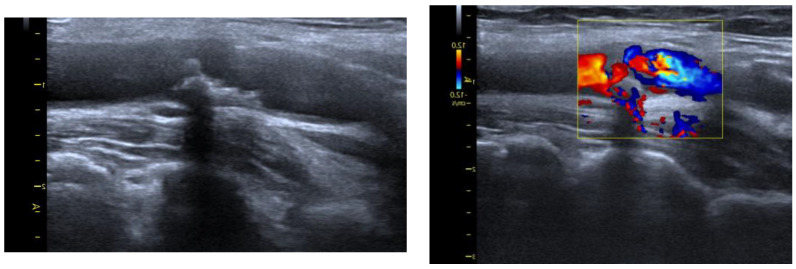
Ultrasound protocol.

The examination of the carotid arteries and assessment of complex intima media thickness (C-IMT) in the extracranial segment was performed using B-mode ultrasound with a linear probe at a frequency of 7.5–10 MHz. According to the Atherosclerosis Risk in Communities Study (ARIC) [95], the C-IMT was evaluated 3 times, and the mean score was taken into consideration. The measurement was performed in the distal common carotid (1 cm proximal to the carotid bulb). For confirmation of atherosclerotic plaque in the carotid artery, we assumed the thickness of the C-IMT complex was >1.5 mm or the presence of plaque, in accordance with the guidelines (Figure 3). We decided to use Carotid MRI to look at the structure of atherosclerotic lesions when there were signs of ruptured plaques, such as fibrofatty; intraplaque hemorrhagic; echolucent appearance; irregular surface.

### 4.3. MRI Protocol

**Figure 4:**
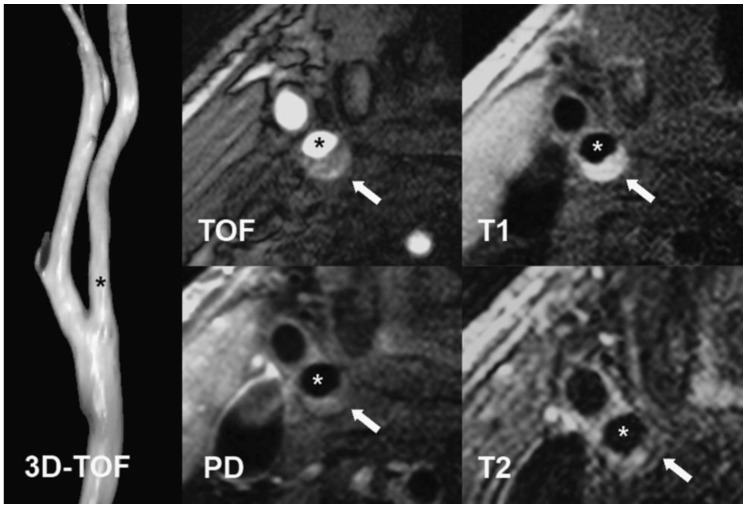
Carotid MRI protocol.

Sixteen participants were investigated with Carotid MRI. The examination was performed on a 1.5 T scanner (General Electric OPTIMA 450 w) with a HeadNeck 8-channel coil. The protocol included TOF (time-of-flight), T2-weighted (2D FIESTA), and T1-weighted (3D TRICKS) sequences, with dynamic contrast enhancement at the following parameters: 1. 2D FIESTA sequence in axial, coronal and sagittal planes at slice thickness = 4 mm with spacing = 1 mm, TR/TE = 4.4/min full, flip angle = 70°, matrix 224 × 320, NEX = 1.0. 2. 3D TOF in axial plane at slice thickness = 2.4 mm with overlap = 1.2 mm, TR/TE = min full/min full, flip angle = 35°, matrix 384 × 256, NEX = 1.0. Dynamic contrast enhanced (DCE) images were acquired with intravenous injection (0.2 mmol/kg) gadolinium contrast (Prohance) using the TRICKS sequence with parameters 3. 3D TRICKS in coronal plane at slice thickness = 2.2 mm, TR/TE = 3.7/min full, flip angle = 20°, matrix 352 × 224, NEX = 0.75. Total scan time was up to 40 min. Based on MRI results, participants were divided into subgroups according to AHA [65] modified criteria, and 10 were in the IV–V class. This group consists of plaques with a lipid-rich or necrotic core, surrounded by fibrous tissue with possible calcification. Moreover, 6 subjects were classified into the VI category, which includes plaques with possible surface defects, IPH, or thrombus. Carotid MRI was performed only once, before the start of the pharmacotherapy (Figure 4).

### 4.4. Serum Arteriosclerotic Markers Analysis

The samples of venous blood were assembled twice, before starting therapy and after 90 days. The blood was collected after an overnight 12 h fasting at 8 a.m. Plasma lipids were assayed using routine laboratory techniques, and LDL levels were measured directly. Plasma levels of interleukins, cytokines, and metalloproteinases were determined using commercially available enzyme immunoassay kits from Cloud-Clone Corp., Houston, TX, USA (Human CD40L—SEA064Hu 98, L170622821; MPO—SEA100Hu 94, L190730464); Diaclone, Besancon, France (Human OPG ELISA Kit—950.030.091; Human OPN Elisa Kit—950.090.094); and BioVendor R&D, Brno, Czech Republic (Human MMP-9—RD191439100CS), respectively. All laboratory tests were conducted on the control group as well. Each experiment was performed on a single sample aliquot to prevent the freeze–thaw effect.

### 4.5. Statistical Analysis

The collected data were processed via the Statistica TIBCO Software Inc., Palo Alto, CA, USA, (2017) version 13.3 program, licensed by the Medical University of Silesia in Katowice. We used the Shapiro–Wilk test to assess the normality of distributions. To fit a normal distribution curve, a log transformation was used for the non-normal variables to fit a normal distribution curve. To compare quantitative variables, the *t*-test for independent means and the *t*-test for dependent means were used. A Student’s paired *t*-test was used to compare the means of variables within the same treatment group. For categorical variables, χ^2^ test was used. In the case of non-compliance with the condition of the parametric ANOVA test, its nonparametric equivalent, the ANOVA Kruskal–Wallis test, was used. We assumed a *p*-value of less than 0.05 was statistically significant.

## 5. Conclusions

PCSK9 inhibitor therapy decreases the concentrations of metalloproteinase 9, osteo-pontin, and osteoprotegerin. Additionally, significant differences in the results of OPN, OPG, and MMP 9 depending on the type of atherosclerotic plaque in the MRI assay were observed. In atherosclerotic plaques considered more stable, the levels of OPN and OPG were higher, while the levels of MMP-9 concentration correlated with the instability of the atherosclerotic plaque. Additional research is required to definitively assess the effect of the novel lipid-lowering therapy on the levels of atherosclerotic biomarkers and plaque rupture risk.

## Figures and Tables

**Table 1:** Correlation of plasma PCSK9 levels with inflammatory markers in patients with no lipid-lowering therapy (ACS, acute coronary syndrome; CAD, coronary artery disease; hs-CRP, high-sensitive C-reactive protein; WBC, white blood cell count).

Author	Study Design	Inflammatory Marker	Coefficient (r)	*p*-Value
Gencer et al. [44]/SPUM-ACS study	Multi-centre prospective cohort study (2168 ACS patients)	hs-CRP	0.077	0.006
Li et al. [45]	Single-centre cross-sectional study (251 stable CAD patients)	WBC	0.167	0.008
Zhang et al. [46]	Cross-sectional study (219 stable CAD patients)	Fibrinogen hs-CRP	0.211 0.153	0.002 0.023
Li et al. [47]	Prospective study (552 CAD patients)	WBC Fibrinogen hs-CRP	0.077 0.181 0.101	0.014 <0.001 0.003

**Table 2:** Baseline characteristics of patients (values are mean ± SD unless indicated otherwise).

	Control Group	Study Group
Number of patients	12	16
Age, years	57 ± 5	58 ± 6
Body mass index	27.2 ± 2.6	27.8 ± 2.0
Women, %	37	38
BMI	27.4 ± 2.7	28.1 ± 2.2
WHO guidelines on physical activity, %	84	81
Smokers, %	26	25
Alcohol abuse	No	No
Systolic blood pressure, mmHg	132 ± 6	134 ± 5
Diastolic blood pressure, mmHg	84 ± 4	83 ± 4
White blood cell count, ×10^9^/L	5.2 ± 1.1	8.0 ± 1.4
High-sensitivity C-reactive protein, mg/L	1.86 ± 0.96	2.84 ± 1.14

**Table 3:** Concentrations of plasma lipids and cytokines in study group before treatment versus control group (values are mean ± SD unless indicated otherwise).

	Control Group	Study Group	*p* Value
Total cholesterol, mg/dL	158.2 ± 10.6	242.7 ± 11.8	*p* < 0.001
Low-density lipoprotein cholesterol, mg/dL	94.4 ± 8.7	181.2 ± 10.2	*p* < 0.001
High-density lipoprotein cholesterol, mg/dL	47.1 ± 4.4	46.1 ± 4.3	*p* < 0.001
Triglicerydes, mg/dL	112.2 ± 9.6	198.6 ± 13.2	*p* < 0.001
Osteopontin, ng/mL	11.12 ± 4.30	15.32 ± 3.20	*p* < 0.01
Osteoprotegerin, pmol/L	4.23 ± 1.20	5.28 ± 1.11	*p* < 0.01
Metaloproteinase-9, ng/mL	255 ± 86	428 ± 82	*p* < 0.05
Soluble CD40 ligand, ng/mL	2.14 ± 0.80	3.69 ± 0.69	*p* > 0.05
Myeloperoxidase, ng/mL	426 ± 112	560 ± 96	*p* > 0.05

**Table 4:** Comparison of arteriosclerotic marker levels between the study group before and after treatment by PCSK9 inhibitors. sCD40L—soluble CD40 ligand; OPN—osteopontin; OPG—osteoprotegerin; MMP-9—metalloproteinase 9; MPO—myeloperoxidase.

Marker	Before Treatment	After Treatment	*p* Value
sCD40L (ng/mL)	3.69 ± 0.9	3.11 ± 0.55	*p* = 0.094
OPN (ng/mL)	15.32 ± 3.20	13.24 ± 3.18	*p* < 0.05
OPG (pmol/L)	5.28 ± 1.11	4.28 ± 1.02	*p* < 0.05
MMP 9 (ng/mL)	428 ± 82	362 ± 64	*p* < 0.01
MPO (ng/mL)	560 ± 96	460 ± 82	*p* = 0.082

**Table 5:** Correlation of concentration arteriosclerotic markers with results of carotid arteries MRI examination. sCD40L—soluble CD40 ligand; OPN—osteopontin; OPG—osteoprotegerin; MMP-9—metalloproteinase 9; MPO—myeloperoxidase.

Type of Carotid Atherosclerotic Lesions:				
Marker	Type IV-V (n = 10)	Type VI (n = 6)	Control Group (n = 12)	*p* Value
OPN (ng/mL)	15.86 ± 3.42	14.94 ± 3.02	11.12 ± 4.30	*p* < 0.01
OPG (pmol/L)	5.64 ± 1.18	5.02 ± 1.0	4.23 ± 1.20	*p* < 0.01
MMP-9 (ng/mL)	398 ± 60	468 ± 94	255 ± 86	*p* < 0.05
CD40L (ng/mL)	3.56 ± 0.62	3.81 ± 0.72	2.14 ± 0.80	*p* > 0.05
MPO (ng/mL)	522 ± 63	592 ± 104	426 ± 112	*p* > 0.05

## Data Availability

The data that support the findings of this study are available from the corresponding author (mbasiak@sum.edu.pl) on reasonable request.
